# Morbidity Patterns of Preschool-age Children

**Published:** 2018-09

**Authors:** Maryan PITYN, Viktoria PASICHNYK, Yaroslav GALAN, Valeryi MELNYK, Zoryana SEMERYAK

**Affiliations:** 1. Lviv State University of Physical Culture, Lviv, Ukraine; 2. Yuriy Fedkovych Chernivtsi National University, Chernivtsi, Ukraine

## Dear Editor-in-Chief

Children’s health is an integral indicator of the general well-being of the society and at the same time is an indicator of all social and environmental problems (1). High stressfulness of socioeconomic factors, negative environmental factors, as well as adverse demographic processes in the society significantly reduce the living standards of the population of our country. The life of people nowadays is accompanied by a sharp worsening of the physical, somatic and psychosocial health (2). Especially serious anxiety is evoked by the health state of modern preschool-age children in view of the increase in pathological changes (3). The period of pre-school children is an important stage for formation and preservation of health in the future.

Protection of children’s health and ensuring the conditions for children comprehensive development are defined in Ukraine as a nationwide priority. One of the most important criteria for assessing health situation is the incidence rate (first discovered diseases) and prevalence rate (all diseases) (3). The indicators of physical development (anthropometric data, pace and peculiarities of their changes in the process of growth, harmonious development, correlation of calendar and biological age, etc.) are the most important health parameters and critical indicators of social well-being of the society (4, 5).

The study of the morbidity patterns of preschool-age children was conducted on the basis of pre-school educational institutions of the city of Lviv and Lviv region in 2017. Overall, 1672 children of preschool-age participated in the study.

Taking into consideration the decrease in the number of children (8003281 children of 0–17 yr of age in 2011, compared with 7181512 in 2015), the incidence rate and prevalence rate remain high (4).

During the study of pre-school educational institutions in Lviv and Lviv region, we studied the number of missed days due to a disease during the year. We have found out that in 2016, out of the total number of children, 16722843 cases of a disease were recorded. The structure of the general morbidity pattern is formed by the following diseases: ARVI(acute respiratory viral infections), 71.6%, bronchitis, 16.3%, tracheitis, 1.7%, pneumonia, 0.9%, otitis, 0.5%, influenza, 1.5%, angina, 0.4%, tonsillitis, 0.5%, conjunctivitis, 0.4%, chicken pox, 1.8%, stomatitis, 0.3%, other diseases, 4.1%.

The resistance of the organism to adverse factors is assessed by the number and duration of the diseases that the child suffered in the previous year. We have found out the number of missed days due to illness and the number of cases of illnesses throughout the year among children of preschool age (3–6 yr of age) (Table 1).

**Table 1:** Morbidity Patterns of Preschool-Age Children

***Age of children***	***Number of children***	***Number of missed days due to a disease per year***	***Number of cases of a disease per year***
3–4 yr of age (Junior preschool age)	550	9,900	1,045
4–5 yr of age (Middle preschool age))	576	8,640	979
5–6 yr of age (**Senior** preschool age)	546	7,098	819

The largest quantity of missed days due to illness was noted in children of younger preschool age (3–4 yr of age), and the smallest number - in children of the senior preschool age (5–6 yr of age).

Having analyzed the received information, we should emphasize the following: there were 1.5 cases of disease per one child of the senior preschool-age and there were 13 d missed because of disease by one child of this age category; among children of the medium preschool age there were 1.7 cases of disease per one child, and there were 15 d missed because of disease per year; concerning junior-schoolers, there were 1.9 cases of disease and 18 missed days per child.

Apart from analysis of the number of missed days and the number of cases due to the disease, the general morbidity of the studied group of children was determined. In children of junior preschool age the general morbidity was 1700 cases per 1000 children of the corresponding age, among children of middle preschool-age -1798, in senior pre-school-age children -1500 cases. The obtained indicators of the general morbidity essentially correspond to the average indicators for Ukraine.
